# Functional interaction between Wnt and Bmp signaling in periosteal bone growth

**DOI:** 10.1038/s41598-021-90324-1

**Published:** 2021-05-24

**Authors:** Deye Song, Guangxu He, Yu Shi, Jiangdong Ni, Fanxin Long

**Affiliations:** 1 Department of Orthopedics, The Second Xiangya Hospital, Central South University, Changsha, 410011 Hunan China; 2 Department of Orthopedic Surgery, Washington University School of Medicine, St. Louis, MO USA; 3 State Key Laboratory of Oral Diseases and National Clinical Research Center for Oral Diseases, West China Hospital of Stomatology, Sichuan University, Chengdu, China; 4 Translational Research Program in Pediatric Orthopedics, Department of Surgery, The Children’s Hospital of Philadelphia, Philadelphia, PA USA; 5 Department of Orthopedic Surgery, University of Pennsylvania, Philadelphia, PA USA

**Keywords:** Developmental biology, Genetics, Physiology

## Abstract

Wnt and Bmp proteins are well known to regulate bone development and homeostasis. Although both signals are extensively studied, their potential interaction in vivo is less well understood. Previous studies have shown that deletion of Bmpr1a, a type I receptor for Bmp signaling, results in excessive trabecular bone formation while diminishing periosteal bone growth. Moreover, forced-expression of the Wnt antagonist Sost suppresses the overgrowth of trabecular bone caused by Bmpr1a deletion, thus implicating hyperactive Wnt signaling in the excessive trabecular bone formation. However, it remains uncertain whether Wnt and Bmp signaling interacts in regulating the periosteal bone growth. Here we show that multiple Wnt genes are markedly suppressed in the cortical bone without Bmpr1a. Importantly, overexpression of Wnt7b fully rescues periosteal bone growth in the Bmpr1a-deficient mice. Thus, pharmacological activation of Wnt signaling can restore normal bone size without intact Bmp signaling.

## Introduction

Bone morphogenetic proteins (Bmp) critically regulates both embryonic development and postnatal tissue homeostasis in mammals^1–3^. Selective deletion of Bmp2 in the limb mesenchyme greatly diminishes bone strength leading to spontaneous fractures in postnatal mice^4^. Deletion of Bmpr1a or Smad4 in mature osteoblasts initially decreases cancellous bone formation, but causes bone resorption deficiency in older mice^5,6^. Interestingly, deletion of Bmpr1a with either Col1-Cre^ER^ or Dmp1-Cre greatly increases cancellous bone mass, whereas deletion with Dmp1-Cre also diminishes periosteal bone growth^7–10^.

In Bmp signaling, dimeric Bmp proteins bind to a hetero-tetramer of serine/threonine kinase receptors composed of two type I (Bmpr1a, Bmpr1b, Acvrl1, Acvr1) and two type II receptors (Bmpr2, Acvr2a, Acvr2b), leading to phosphorylation and activation of the type I receptor by the type II receptor which has constitutively active kinase activity^11,12^. The activated type I receptors then activate multiple downstream mechanisms including the Smad pathway, TAK1-p38 or PI3K-Akt signaling axis^3,12–16^. More recently, Bmp signaling has been shown to activate both Smad and mTORC1 signaling to regulate osteoblast differentiation and bone formation^10,17^. Thus, depending on the cellular context, Bmp may employ different effectors to control various biological processes.

Wnt proteins, a family of secreted glycoproteins, have been shown to stimulate bone formation. In the best studied mechanism, Wnt proteins signal through frizzled proteins and the low-density lipoprotein receptor-related protein Lrp5 or Lrp6 to stabilize ß-catenin which in turn activates gene expression^18^. Wnt signaling is tempered by various secreted antagonists including sclerostin (encoded by the SOST gene) that compete with Wnt proteins for binding to Lrp5/6^19^. In humans, inhibitory mutations of LRP5 cause osteoporosis, whereas activating mutations of LRP5 or loss of SOST results in osteosclerosis^20–24^. An antibody against sclerostin was recently approved by FDA for treatment of severe postmenopausal osteoporosis.

The role of Lrp5 in bone formation is conserved between mice and human, as Lrp5 knockout mice develop osteopenia whereas mice carrying the Lrp5 hyperactive mutations (e.g., A214V) exhibit osteosclerosis^25–27^. Consistent with the paradigm that Lrp5 and Lrp6 mediates Wnt signaling to stabilize β-catenin, numerous genetic studies have demonstrated that β-catenin critically controls osteoblast differentiation in the mouse embryo^28–31^. Postnatal deletion of β-catenin in osteoblast-lineage cells suppresses osteoblast differentiation, diminishes osteoblast activity and reduces osteoblast lifespan^32,33^. Besides β-catenin signaling, Wnt proteins also activate mTORC1 signaling to stimulate bone formation^34^. Upon activation of mTORC1 signaling, Wnt has been shown to stimulate glutaminolysis to meet the anabolic needs of osteoblasts^35^. In addition, Wnt acutely activates glycolysis through mTORC2 signaling^36^. Thus, Wnt signaling exerts the bone anabolic function through multiple intracellular mechanisms. Despite the extensive studies of Wnt signaling in bone, how Wnt interacts with Bmp in osteogenic regulation is less well explored. Previous experiments regarding the two signals in osteoblast differentiation in vitro have yielded disparate results, likely due to differences in the cellular context^17,37^.

To address the relationship between Bmp and Wnt signaling in vivo, we have previously shown that deletion of Bmpr1a by Dmp1-Cre leads to downregulation of Sost expression in osteocytes, and that forced-expression of Sost in the Bmpr1a-deficient mice partially corrected the hyperproliferation of preosteoblasts in trabecular bone^38^. Thus, Bmp signaling through Bmpr1a normally limits Wnt signaling via the induction of Sost to restrict preosteoblast proliferation during trabecular bone formation.

Here we report that multiple Wnt ligands are notably reduced in the cortical bone of Bmpr1a-deficient mice. Importantly, overexpression of Wnt7b restores the normal bone size by boosting osteoblast activity in the face of Bmpr1a deficiency. The results support a model wherein Bmp signaling via Bmpr1a induces Wnt expression to promote periosteal bone growth.

## Results

### Loss of Bmpr1a diminishes bone anabolic Wnt expression in cortical bone

**Figure 1:**
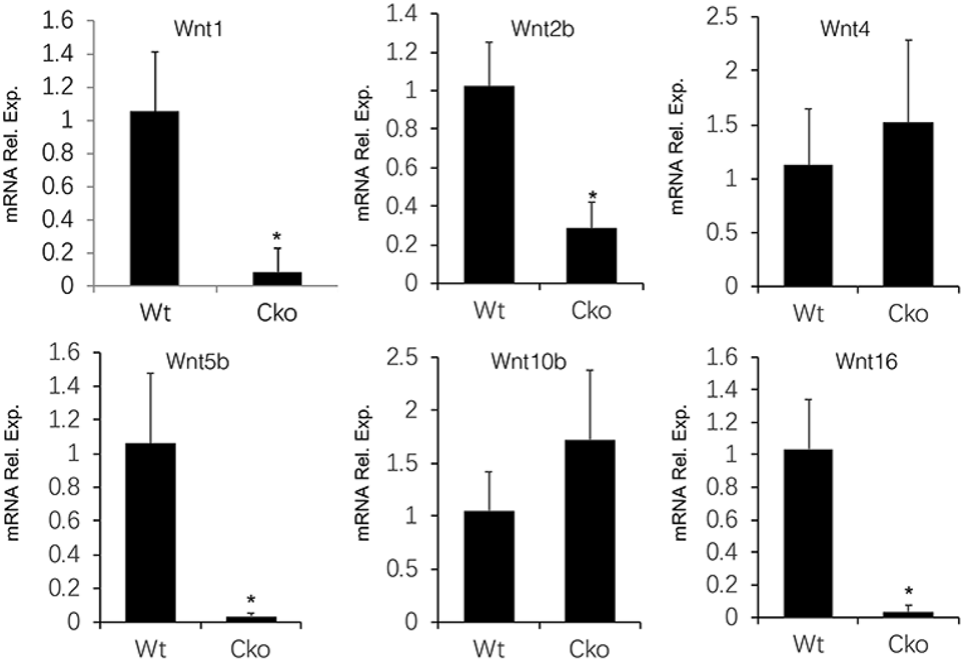
Bmpr1a deletion diminishes Wnt expression in cortical bone. Relative mRNA levels of Wnt genes determined by RT-qPCR in the cortical bone of wild type (WT) versus Dmp1-Cre; Bmpr1a^f/f^ (CKO) male littermates at P33. *: *P* < 0.05, Student’s t-test, n = 3.

To assess the potential effect of Bmp signaling on Wnt expression, we surveyed the mRNA levels of major Wnt ligands in the long bones of wild type versus Dmp1-Cre; Bmpr1a (Bmpr1a CKO) littermate mice. We selected the Wnt ligands with RPKM > 1 in a previous RNA-seq study of the normal cortical bone, but also included Wnt1 and Wnt7b known to stimulate bone growth^34,39–43^. RT-qPCR experiments with RNA extracted from the tibial and femoral diaphysis showed that Wnt1, Wnt2b, Wnt5b and Wnt16 were significantly reduced in the Bmpr1a CKO versus wild type samples, whereas Wnt4 and Wnt10b were not affected and Wnt7b was below the detection level in both samples (Ct > 40) (Fig. 1). As both Wnt1 and Wnt16 have been shown to promote bone formation, the data indicate that downregulation of the bone anabolic Wnt ligands may contribute to the cortical bone defect in the Bmpr1a deficient mice^41,44,45^.

### Overexpression of Wnt7b rescues bone size in Bmpr1a-deficient mice

**Figure 2:**
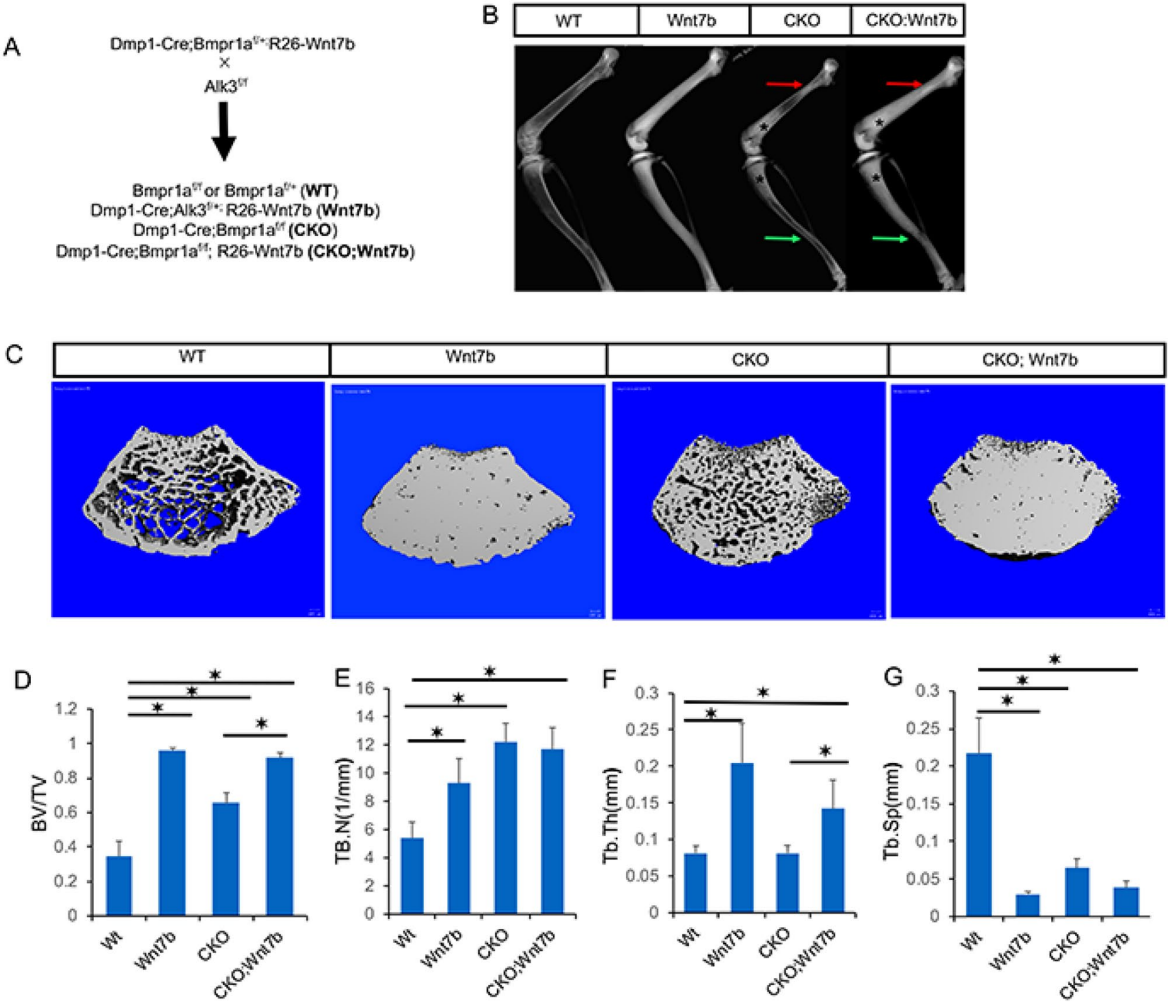
Overexpression of Wnt7b increases trabecular bone mass in both normal and Bmpr1a CKO background. (**A**) Mating scheme. (**B**) Representative X-ray radiography of the hindlimbs from littermate mice at P33. Arrows denote areas of reduced bone size (red: proximal femur; green: tibiofibular junction) in CKO but corrected in CKO; Wnt7b mice. (C) Representative μCT 3D reconstruction images of the distal metaphysis of the femur in littermate mice. (**D**–**G**) μCT quantification of cancellous bone parameters in the distal metaphysis of the femur. *P < 0.05, two-way ANOVA, n = 6 (4 females, 2 males).

Although Wnt7b is normally expressed at a low level in the cortical bone, overexpression of the protein has been shown to stimulate bone growth at the periosteal surface^46^. Considering that Wnt1 and Wnt16, both physiological regulators of the cortical bone, were significantly reduced in the Bmpr1a CKO mice, we reasoned that overexpression of Wnt7b might compensate for the loss of the endogenous ligands and thus normalize the bone size. To test this hypothesis, we created compound mutant animals with the genotype of Dmp1-Cre; Bmpr1a^f/f^; R26-Wnt7b (CKO, Wnt7b) to compare with the Dmp1-Cre; Bmpr1a^f/f^ littermates (CKO) (Fig. 2A). The same cross also produced normal mice with the genotype of Bmpr1a^f/f^ or Bmpr1a^f/+^ (WT), and Wnt7b-overexpressing mice with the genotype of Dmp1-Cre; Bmpr1a^f/+^; R26-Wnt7b (Wnt7b). Mice of all genotypes appeared healthy without any overt abnormality.

The mice were subjected to X-ray-based imaging at 33 days of age. As expected from earlier studies, contact radiography revealed a marked increase in bone density in the Wnt7b-overexpressing mice (Wnt7b) compared to the normal control (WT) (Fig. 2B, left two images)^34^. Also confirming previous results, the Bmpr1a CKO mice exhibited a marked increase in trabecular bone, but a clear reduction in the cross-sectional size of the tibia and the proximal femur (Fig. 2B, CKO vs WT)^10^. Overexpression of Wnt7b in the background of CKO further increased trabecular bone mass (Fig. 2B, CKO; Wnt7b vs CKO, asterisks). µCT imaging of the distal femur trabecular bone confirmed that Wnt7b overexpression markedly increased the trabecular bone mass in both normal and CKO background (Fig. 2C). Quantification of the µCT data showed that overexpression of Wnt7b essentially maximized the trabecular bone mass regardless of Bmpr1a deletion (Fig. 2D). µCT quantification also revealed that in the normal background Wnt7b increased both trabecular number and thickness whereas deletion of Bmpr1a (CKO) increased trabecular number only (Fig. 2E,F). In the CKO background, however, Wnt7b increased only trabecular thickness likely due to the already high trabecular number (Fig. 2E,F). In keeping with the increase in trabecular number and thickness, trabecular separation was decreased in all mutant mice compared to the wild type (Fig. 2G). Thus, Wnt7b greatly enhances trabecular bone formation regardless of intact Bmp signaling.

**Figure 3:**
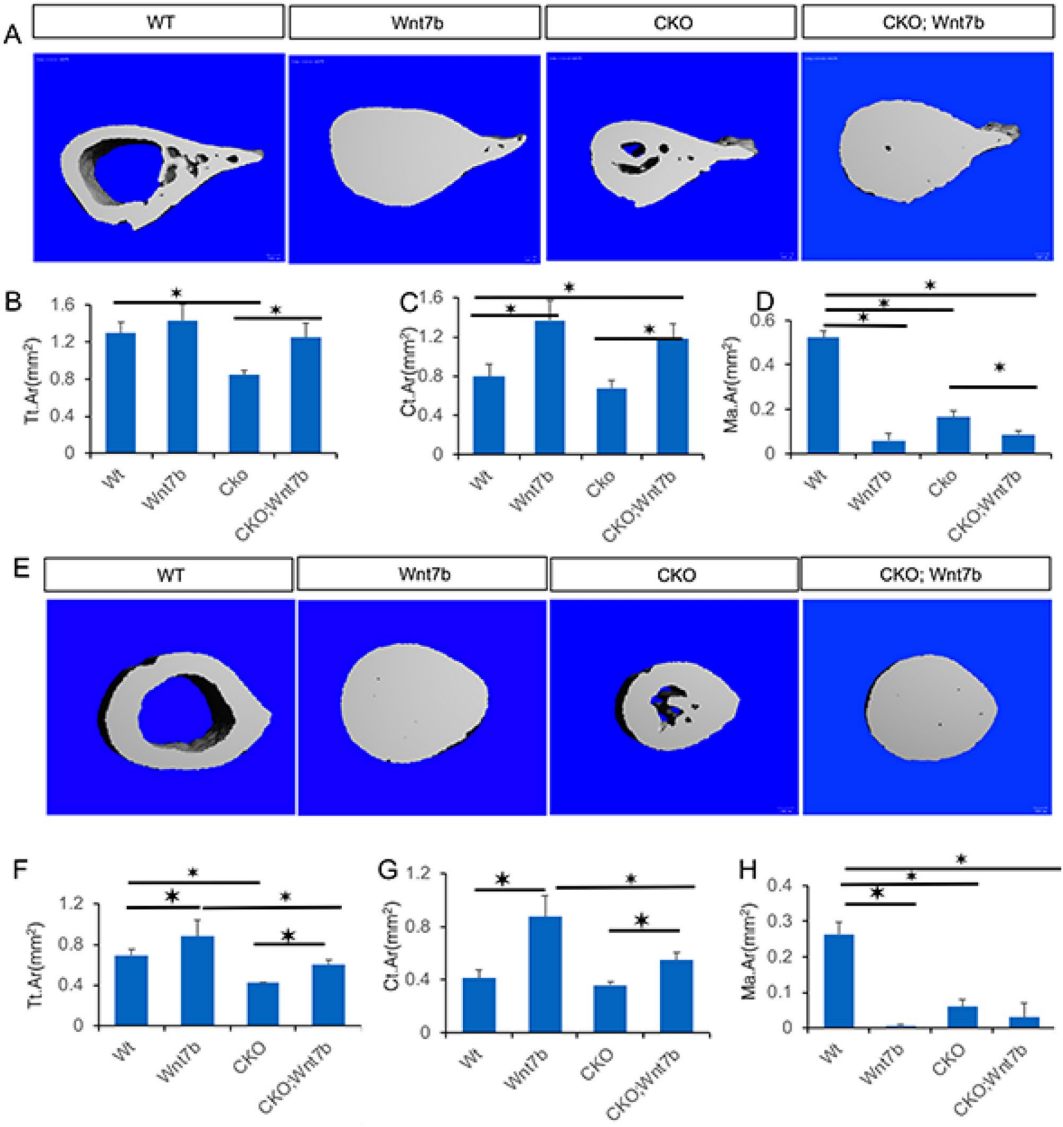
Overexpression of Wnt7b rescues diminished bone size in Bmpr1a-deficient mice. (**A**) Representative 3D reconstruction of cross-sectional μCT images at the proximal femur in littermate mice at P33. (**B**–**D**) μCT quantification of cortical bone parameters in the proximal femur. *: *P* < 0.05, two-way ANOVA, n = 5 (4 females, 1 male). (**E**) Representative 3D reconstruction of cross-sectional μCT images of the tibia immediately above the tibiofibular junction. (**F**–**H**) μCT quantification of cortical bone parameters of the tibia immediately above tibiofibular junction. *: *P* < 0.05, two-way ANOVA, n = 6 (4 females, 2 males).

X-ray imaging also detected the impact of Wnt7b overexpression on bone size. X-ray contact radiography revealed that Wnt7b increased both tibial and femoral width in CKO (Fig. 2B, arrows). Further analyses of the proximal femur by µCT confirmed that the overall cross-sectional size was notably increased in the CKO; Wnt7b mice compared to CKO (Fig. 3A). Quantification of the µCT data confirmed that the total area (Tt. Ar) across the proximal femur was fully recovered to the normal size (WT or Wnt7b) in the CKO; Wnt7b mice from the deficit seen in CKO (Fig. 3B). Wnt7b also markedly increased the cortical bone area (Ct. Ar) and all but eliminated the marrow area (Ma. Ar) in both normal (WT) and CKO background (Fig. 3C,D). Similarly, µCT analysis of the tibia at the tibiofibular junction showed that Wnt7b restored the cross-sectional size (Tt. Ar) in CKO; Wnt7b mice to the normal level (WT) (Fig. 3E,F). However, here Wnt7b overexpression increased the cortical bone size above normal although it did not do so in the proximal femur, thus highlighting regional differences in the anabolic response (Fig. 3F). Like in the proximal femur, Wnt7b markedly increased the cortical bone area (Ct. Ar) and essentially eliminated the marrow space (Ma. Ar) in the tibia (Fig. 3G,H). Thus, Wnt7b overexpression not only increases trabecular and endosteal bone but also fully restores periosteal bone growth in the absence of Bmpr1a.

### Wnt7b overexpression restricts cell proliferation in trabecular bone region

**Figure 4:**
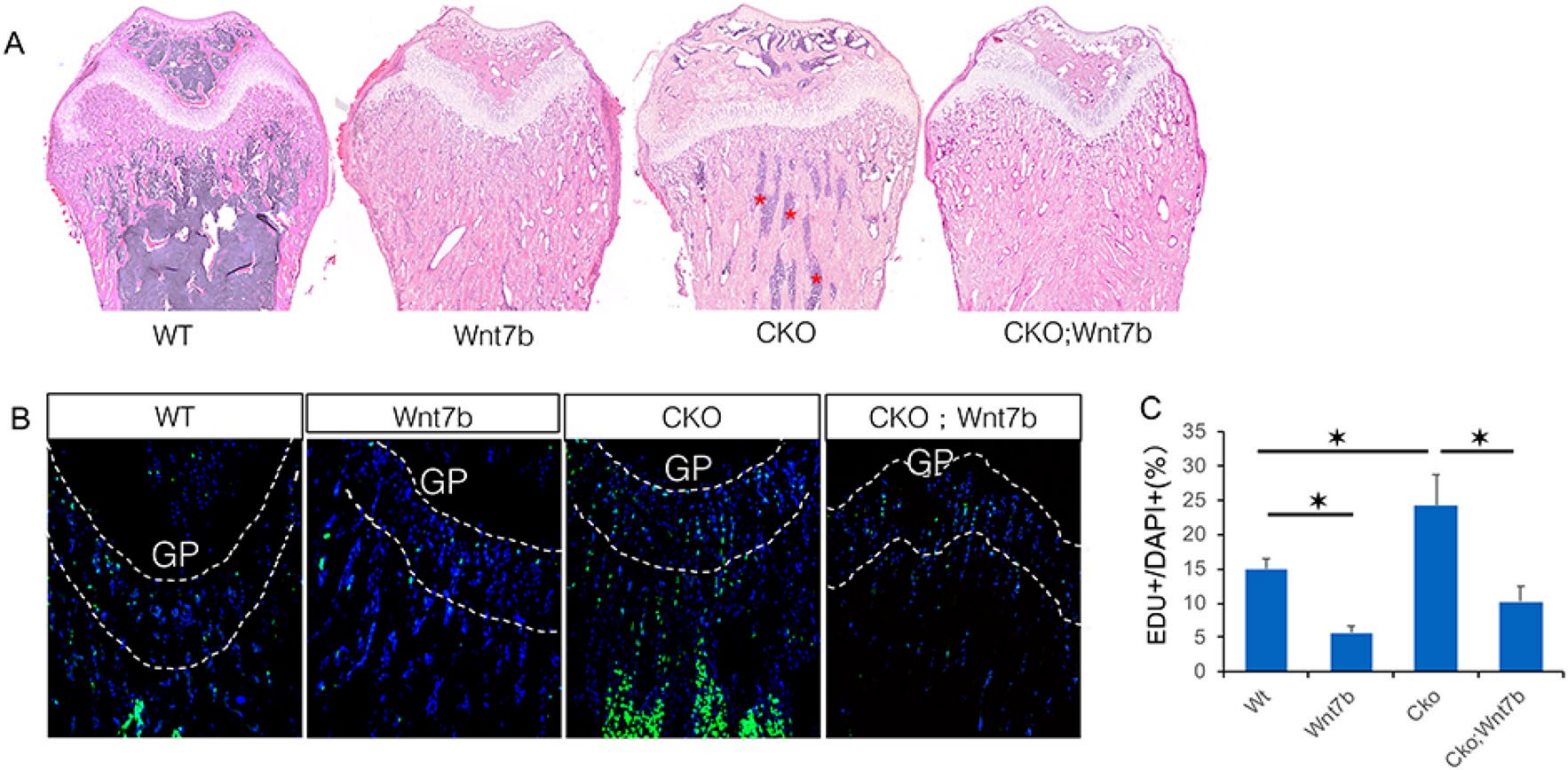
Wnt7b overexpression reduces cell proliferation in trabecular bone region. (**A**) Representative images of H&E-stained sections of the distal femur at P33. (**B**) Representative images of the distal femur labeled with EdU at P33. EdU signal is in green and DAPI nuclei staining in blue. (**C**) EdU labeling index at the chondro-osseous junction of distal femur (100 µm region immediately below the growth plate). *: *P* < 0.05, two-way ANOVA, n = 3 (females). GP: growth plate.

We next examined the trabecular bone in more detail. H&E staining showed that Wnt7b overexpression in either normal or CKO background (Wnt7b or CKO; Wnt7b, respectively) caused massive bone buildup and complete preclusion of the hematopoietic marrow cells in the distal femur (Fig. 4A). Deletion of Bmpr1a, as previously reported, also caused severe osteosclerosis leaving only residual marrow cells present in the region (Fig. 4A, CKO)^10,38^. Thus, histology confirms that Wnt7b overexpression intensifies the osteosclerotic phenotype caused by Bmpr1a deletion.

As previous studies have shown that Bmpr1a deletion increases preosteoblast proliferation as a main cause for osteosclerosis, we set out to determine whether Wnt7b has a similar effect by performing EdU labeling experiments. Remarkably, Wnt7b overexpression in the normal background greatly reduced the proliferation rate within the chondro-osseous junction where preosteoblasts were highly enriched (Fig. 4B,C, Wnt7b vs WT). Moreover, Wnt7b completely neutralized the hyperproliferation caused by Bmpr1a deletion. (Fig. 4B,C, CKO; Wnt7b vs CKO). Therefore, Wnt7b increases trabecular bone formation through a mechanism distinct from stimulation of cell proliferation, likely via increased osteoblast differentiation and activity.

### Wnt7b stimulates periosteal bone growth in the absence of Bmpr1a

**Figure 5:**
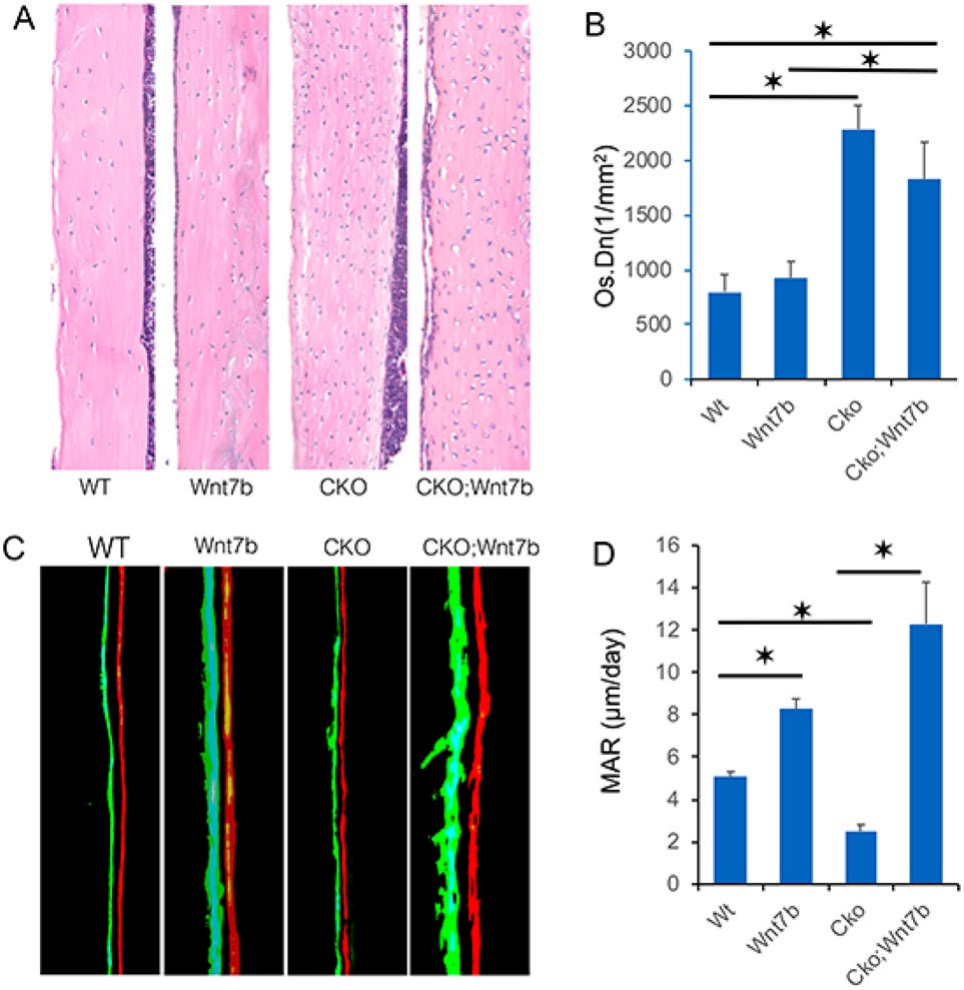
Overexpression of Wnt7b restores periosteal bone growth in Bmpr1a-deficient mice. (**A**) Representative H&E staining of the femoral cortical bone in littermate mice at P33. (**B**) Quantification of osteocyte density in the cortical bone. *P < 0.05, two-way ANOVA, n = 3 (females). (**C**) Representative images of calcein and alizarin double labeling at periosteal surface littermate mice at P33. (**D**) Quantification of mineral apposition rate (MAR). *: *P* < 0.05, two-way ANOVA, n = 3 (females).

To gain more insight about the effect of Wnt7b on the cortical bone, we performed histomorphometry with mice of the different genotypes. By H&E staining of longitudinal sections through the femur, Wnt7b overexpression in the normal background did not cause an increase in osteocyte density as seen in the CKO mouse (Fig. 5A,B). Wnt7b appeared to moderate the high osteocyte density in the CKO; Wnt7b compound mutant compared to CKO, but quantification showed that the downward trend did not reach statistical significance (Fig. 5A,B). The bone formation activity was monitored by double labeling with calcein followed by alizarin red. Wnt7b notably increased whereas Bmpr1a deletion (CKO) decreased the mineral apposition rate (MAR) at the periosteal bone surface when compared to normal (WT) (Fig. 5C,D). Importantly, MAR in the CKO; Wnt7b mouse was markedly higher than that in the CKO or WT mice (Fig. 5C,D). Overall, pharmacological overexpression of Wnt7b fully restored periosteal bone growth in the face of Bmpr1a deletion.

## Discussion

We have shown that deletion of Bmpr1a in osteoblasts and osteocytes notably suppressed the expression of multiple bone anabolic Wnt genes in the cortical bone. Furthermore, overexpression of Wnt7b in the same cells fully rescued the defect in periosteal bone growth caused by Bmpr1a deletion. We have previously shown that a hyperactive form of Lrp5 (A214V) failed to correct the bone size deficit in the Bmpr1a-deficient background, raising the possibility that Bmp signaling might regulate periosteal growth independent of Wnt^38^. However, as signaling by Lrp5 (A214V) requires the binding of Wnt proteins, its activity is likely to be muted when the endogenous Wnt levels are diminished in the Bmpr1a CKO bones as shown here. Thus, the current study lends support to the alternative model that Bmp signaling stimulates periosteal bone growth partly through induction of Wnt expression in the cortical bone.

This and our previous study have shed light on the complexity of interaction between Wnt and Bmp in trabecular versus cortical bone^38^. In both trabecular and cortical bone, Bmpr1a deletion reduces the expression of the Wnt antagonist Sost, but a bone overgrowth phenotype is only seen with the trabecular bone which is at least partially due to increased preosteoblast proliferation and can be rescued by forced-expression of Sost^10,38^. In the cortical bone, the reduced Sost expression paradoxically concurs with diminished periosteal growth. In the current study, we show that Bmpr1a deletion causes a notable decrease in the expression of several Wnt ligands in the cortical bone although they are not examined in the trabecular bone. The functional contribution of those ligands is not tested here, but pharmacological overexpression of Wnt7b is sufficient to overcome the bone size deficit in the Bmpr1a CKO mice. The exact mechanism for Wnt7b to rescue periosteal bone growth remains to be elucidated, but previous studies have shown that Wnt7b overexpression enhances bone formation at least partly by activating mTORC1 in osteoblasts^34^. However, we cannot rule out that Wnt7b overexpression in the Bmpr1a CKO mice may increase Bmp expression which could signal through the remaining type I receptors to activate mTORC1 or Smad signaling^10,17^. Although we previously showed that the bone phenotype in Bmpr1a CKO was not replicated by Smad4 deletion, potential activation of Smad signaling upon Wnt7b overexpression could still contribute to the rescuing effect^10^. Finally, the identity of the Wnt molecules promoting preosteoblast proliferation in the trabecular bone is unknown at present, but it is clearly distinct from those with similar properties to Wnt7b, as Wnt7b overexpression had the opposite effect on cell proliferation in the trabecular region.

Due to its low expression normally in the cortical bone, Wnt7b is unlikely to be the endogenous Wnt ligand responsible for the bone size defect in the Bmpr1a CKO mice. Wnt1 on the other hand is a probable candidate as it was clearly downregulated by Bmpr1a deletion, and has been shown to promote cortical bone growth in both loss- and gain-of-function experiments^41^. Mechanistically, Wnt1, like Wnt7b, has been shown to activate mTORC1 signaling in osteoblasts^34,41^. Wnt16 was also reduced in bone upon Bmpr1a deletion, but it has been implicated mainly in suppressing osteoclastogenesis at the endosteal surface, even though overexpression of Wnt16 also stimulated bone formation while suppressing bone resorption^45,47^. The effect of Wnt16 on bone resorption also appears to diverge from that of Wnt1 or Wnt7b, as overexpression of either molecule increased overall bone resorption in the mouse^34,41^. Future experiments are necessary to determine whether Wnt1 is indeed the physiological ligand whose downregulation by Bmpr1a deletion is compensated by the forced-expression of Wnt7b.

## Materials and methods

### Mouse strains

Dmp1-Cre^48^, Bmpr1a^f/f^^49^, R26-Wnt7b^34^ mouse strains as previously described were maintained in a mixed genetic background of predominantly C57BL6/J. All analyses were conducted with sex-matched littermates including both males and females at 33 days of age (P33). Both males and females were analyzed for all parameters and they showed the same phenotype. The animals were group housed in a specific pathogen free (SPF) barrier facility with a 12-h light cycle (6 am-6 pm) and fed standard chow (PicoLab mouse diet 20, #5058). The Animal Studies Committee at Washington University in St. Louis School of Medicine approved the study. All methods were performed in compliance with relevant guidelines and regulations. The studies were carried out according to the ARRIVE guidelines.

### Bone morphological analyses

X-ray contact radiography was performed with Faxitron (Faxitron X-ray Corp) for 20-s exposures at 25 kV. Micro-computed tomography was conducted with μCT 40 (Scanco Medical AG) and with key parameters as follows: voxel size 10 μm^3^, X-ray tube potential 55 kVp, X-ray intensity 145 μA, integration time 300 ms^50^. Quantitative trabecular bone parameters were assessed with 100 μCT slices (1.6 mm) immediately below the growth plate, with a threshold set at 240. For cortical bone parameters, 50 μCT slices (0.8 mm) at indicated locations were analyzed with a threshold of 260.

Hematoxylin and eosin (H&E) staining was performed on 6-μm paraffin sections. Before sectioning, the bones were fixed overnight with neutral buffered 10% formalin followed by decalcification with daily change of 14% EDTA (pH 7.4) for 2 weeks. For dynamic histomorphometry, calcein (Sigma-Aldrich) and Alizarin red (Sigma-Aldrich) solutions were injected intraperitoneally at 7 and 2 days, respectively, prior to sacrifice. Bones were fixed in 70% ethanol, embedded in methyl-methacrylate and sectioned at 10 μm. Quantifications were done with Bioquant Osteo II from three sections per mouse and three mice for each genotype.

### EdU labeling assay

EdU (Invitrogen) dissolved in water was injected intraperitoneally at 10 μg/g body weight at 4 h before harvest. EdU incorporation was detected by a click reaction according to manufacturer’s instructions (Thermo Fisher Scientific, C10337). Images were acquired with the Nikon C-1 confocal system.

### RT-qPCR

RNA was extracted from femurs and tibiae of P33 mice. After the bones were cleanly dissected and with the epiphysis removed, the marrow was discarded by centrifugation. The cleaned bone shafts were then cut into small pieces and rinsed with ice-cold PBS for three times before being snap-frozen in liquid nitrogen and pulverized at 2000 rpm for 20 s with a Mikro-Dismembrator. RNA was then extracted from the pulverized bone with Trizol (Invitrogen) and purified with the RNeasy RNA extraction kit (Qiagen). 1 µg RNA was used for cDNA synthesis with the iScript cDNA synthesis kit (Bio-Rad). qPCR was performed with SYBR Green Supermix (SsoAdvanced, Bio-Rad) in an ABI StepOne Plus machine. Primer sequences are listed in Table 1. 18S rRNA was used as an internal control for normalization and the relative expression was determined with the 2^−(ΔΔCt)^ method.

**Table 1:** Nucleotide sequences for RT-qPCR primers (5′ to 3′).

Wnt16-F	CAGGGCAACTGGATGTGGTT
Wnt16-R	CTAGGCAGCAGGTACGGTT
Wnt10b-F	GCGGGTCTCCTGTTCTTGG
Wnt10b-R	CCGGGAAGTTTAAGGCCCAG
Wnt2b-F	CCGACGTGTCCCCATCTTC
Wnt2b-R	GCCCCTATGTACCACCAGGA
Wnt7b-F	TTTGGCGTCCTCTACGTGAAG
Wnt7b-R	CCCCGATCACAATGATGGCA
Wnt5b-F	CTGCTGACTGACGCCAACT
Wnt5b-R	CCTGATACAACTGACACAGCTTT
Wnt4-F	AGACGTGCGAGAAACTCAAAG
Wnt4-R	GGAACTGGTATTGGCACTCCT
Wnt1-F	AGCTGGGTTTCTACTACGTTG
Wnt1-R	TCTTGGAATCCGTCAACAGG

### Statistics

Statistical significance was calculated with either Student’s t-test or Two-Way Factorial ANOVA for Independent Samples (vassarstats.net) as indicated in figure legends.
